# Exploring the “Global Workspace” of Consciousness

**DOI:** 10.1371/journal.pbio.1000066

**Published:** 2009-03-17

**Authors:** Richard Robinson

As an explanatory principle in biology, vitalism has long been in decline, as one discovery after another revealed that mechanisms provide convincing explanations—hearts are pumps, genes are code—for all manner of life's phenomena. But even through the 20th century, the mind has been vitalism's last redoubt, because there has been no simple, satisfactory, mechanistic explanation of the most puzzling aspect of the mind: the nature of conscious awareness. For many years, even asking questions about the inner workings of this mental black box was taboo among some groups of scientists.

But that has all changed. A flood of new discoveries in every area of neuroscience has led to competing models of consciousness, and most importantly, testable hypotheses. A new study by Raphael Gaillard, Lionel Naccache, and colleagues provides support for one such model by showing that conscious, but not nonconscious, visual information is rapidly and widely distributed across the brain, provoking the synchronized brain activity that is the hallmark of conscious processing.

The model, called the “global workspace” model, posits that incoming information becomes conscious only when three conditions are met. First, the information must be represented by networks of sensory neurons, such as those in the primary visual cortex at the rear of the brain, that process incoming visual signals. Second, this representation must last long enough to gain access to (“come to the attention of”) a second stage of processing, distributed across the brain's cortex, and especially involving the prefrontal cortex, which is believed to be a major center for associating multiple kinds of information. Third and finally, this combination of bottom-up information propagation and top-down amplification through attention must “ignite” to create a state of reverberating, coherent activity among many different brain centers. That, according to the model, is what we experience as consciousness.

**Figure:**
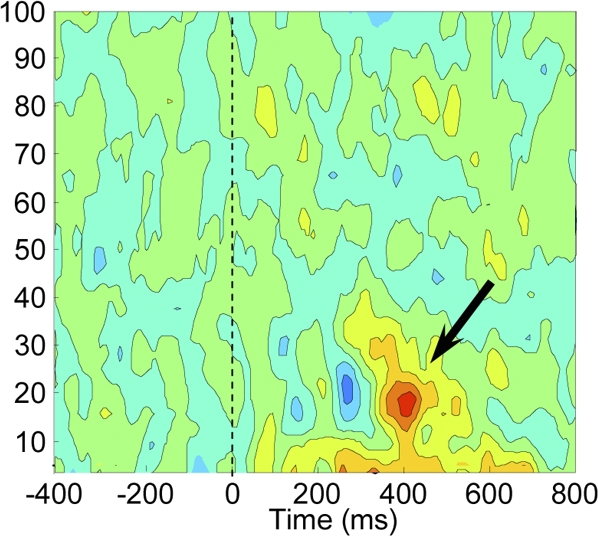
A hallmark of conscious visual perception is the sudden increase of communication between distant brain areas. Less than half a second after a word was flashed on a screen, its conscious perception was associated with a burst of synchrony within a dedicated range of frequencies (red spot around 20 Hz). This signature was absent during nonconscious perception of subliminal words.

A difficulty of consciousness studies is that humans are the best subjects, but probing inside their brains purely for research purposes is unethical. So the authors turned to patients with medically intractable epilepsy, who, in preparation for surgery, had required multiple shallow recording electrodes to be implanted within their cerebral cortexes to locate seizure activity. The authors showed the participants a computer screen, upon which they projected first a set of hatch marks (acting as a meaningless “mask”), then a word, and then either a blank screen or a set of ampersands (another mask). The entire sequence took only half a second, and the word was flashed so briefly that in neither case could the participant name the word. But in both cases the word was registered at the earliest stages of visual processing nonetheless, as shown by electrical activity in the primary visual cortex, thus meeting the first condition of the global workspace model.

The words themselves were either of a threatening (“kill,” “danger”) or nonthreatening (“cousin,” “see”) nature. When participants were exposed to words followed by the second mask, they could guess the nature of the words they saw with no better than chance frequency. The second stage was not reached; the fire was doused before it could ignite.

But when the second mask was absent, the words were consciously reportable and readable, so the authors could compare masked (nonconscious) perception and unmasked (conscious) perception of briefly flashed words.

The electrodes told the same story. There were sustained voltage changes throughout the brain, especially in the prefrontal cortex. The voltage changes were accompanied by an increase in power within specific brain wave frequencies associated with cognitive processing, as well as synchronization of activity among many different brain regions—in short, the brain gave every appearance of thinking about the word. In the language of the global workspace model, the stimulus endured long enough, and gained enough attention, to be promoted to the workspace, at which point information about it was broadcast throughout the brain to be processed in multiple ways—including the determination of its emotional character.

The authors point out that consciousness is always “about” something; there may be no “pure” state of consciousness that is independent of the content of our thought, and so an important question is whether these results, concerning a single word the participant could not even name, are generalizable to understanding the ongoing flow of our conscious experience. Further experiments with other kinds of stimuli may reveal which late-stage, widespread brain events are common to all conscious processing, and which are specific to the experiment at hand.

